# Exploring the Correlation and Protective Role of Diabetes Mellitus in Aortic Aneurysm Disease

**DOI:** 10.3389/fcvm.2021.769343

**Published:** 2021-11-08

**Authors:** Divyatha Arun, Wahaj Munir, Lara Victoria Schmitt, Rohan Vyas, Jeuela Iris Ravindran, Mohamad Bashir, Ian Michael Williams, Bashi Velayudhan, Mohammed Idhrees

**Affiliations:** ^1^Department of Endocrinology, Columbia Asia Referral Hospital, A Unit of Manipal Hospital, Yeshwanthpur, Bengaluru, India; ^2^Barts and the London School of Medicine and Dentistry, Queen Mary University of London, London, United Kingdom; ^3^Institue of Cardiac and Aortic Disorders, SRM Institutes for Medical Science (SIMS Hospitals), Chennai, India; ^4^Vascular Surgery, University Hospital of Wales, Cardiff, United Kingdom

**Keywords:** diabetes mellitus, aorta, aneurysm, dissection, hyperglycemia, insulin resistance, protection

## Abstract

**Introduction:** Diabetes mellitus is recognised as a significant risk factor for cardiovascular and peripheral vascular disease, as the abnormal metabolic state increases the risk for atherosclerosis, occlusive arterial disease and vascular dysfunction. There have been reports of potential association across the literature that illustrates a link between diabetes mellitus and aortic aneurysm, with the former having a protective role on the development of the latter.

**Methods:** A thorough literature search was performed through electronic databases, to provide a comprehensive review of the study's reporting on the association of diabetes mellitus and aortic aneurysm, discussing the mechanisms that have been reported; furthemore, we reviewed the reports of the impact of oral hypoglycameic agents on aortic aneurysms.

**Results:** Various proposed mechanisms are involved in this protective process including endothelial dysfunction, chronic hyperglycemia and insulin resistance. The evidence suggests a negative association between these disease process, with prevelance of diabetes mellitus resulting in lower rates of aortic aneurysm, *via* its protective mechanistic action. The increase in advanced glycation end products, increased arterial stiffness and vascular remodelling seen in diabetes, was found to have a profound impact on aneurysm development, its slow progression and lower rupture rate in these individuals. This review has also highlighted the role of oral hypoglycaemic agents having a protective effect against AA disease.

**Conclusion:** A decrease in development, progression and mortality from aortic aneurysms as well as reduced rates of dissection, have been observed in those with diabetes. This review has provided a comprehensive insight on the effect of diabetes and its physiological processes, and elements of its con-committant treatment, having a protective role against these aortic diseases.

## Introduction

Diabetes mellitus (DM) is one of today's fastest rising healthcare challenges that we are facing, with almost half a billion adults between the ages of 20 and 79 estimated to currently have the disease. This is equivalent to just under 10% of the global population in this given age range. Predictions concerning the rising diabetic population expect a rapid growth to near 600 and 700 million by 2030 and 2045, respectively. In 2019, deaths associated with DM and the consequent complications of the disease were approximated at 4.2 million (1).

Aortic aneurysms (AA), caused by underlying weakness of the aortic wall which subsequently develops into permanent dilation of the aortic lumen, are most commonly asymptomatic until the catastrophic event of rupture and are associated with a mortality rate of 80%. Whilst AA can occur at any level of the thoracic or abdominal aorta, the most common locations are the infrarenal and proximal thoracic regions. AA show a male preponderance with advancing age, smoking, positive family history, hypertension, hyperlipidaemia and atherosclerosis attributed as the other risk factors (2, 3). Abdominal aortic aneurysms (AAA) are responsible for 1–3% of male deaths between the ages of 65 and 85 in developed regions (2, 4, 5).

Whilst there are many similarities in the mechanism of thoracic aortic aneurysms (TAA) and AAA, distinctive features also contrast the two. The complex pathological process leading to AAA formation involves alteration or depletion of vascular smooth muscle cells (VSMC) linked to inflammatory cell infiltration, re-modelling of the extra-cellular matrix (ECM), intraluminal thrombus development and oxidative stress. Changes in the ECM and the tone of VSMC have significant associations with TAA development, which typically occurs at a younger age, is associated with genetic disorders and frequently leads to aortic dissection (AD).

DM represents a major cardiovascular risk factor but numerous epidemiological reports have shown a negative association between DM and both AAA and TAA, thereby conferring a protective effect on them (6, 7). This review aims to summarise the current available knowledge on the underlying mechanisms of this inverse relationship between these disease processes.

## Pathology of Aneurysm Formation

Aneurysm, derived from the Greek term *Aneurysma*, meaning widening, is characterised by permanent irreversible dilatation of a vessel. Conventional diagnosis of AAA requires an aortic diameter of greater than 30 mm, affecting all three layers of the arterial wall (4).

The mechanism of AA formation is complex, with inflammatory cell infiltrate (namely monocytes and macrophages) in the tunica media layer of the aortic wall playing an important role. The macrophage increase results in ECM remodelling which requires a balance between proteases like matrix metalloproteinases (MMPs) and its inhibitors (8). The characteristic dilatation of the aortic wall seen in AA, could partially be due to an imbalanced relation between wall stress and strength. Furthermore, studies have suggested an unmediated association between significant aortic wall stress and AA rupture risk. Aortic wall remodelling is both an expected and vital response to increased wall stress (9–13).

The walls of AAA show widespread inflammation, VSMC depletion and ECM degradation. These changes in the ECM are associated with extensive proteolysis causing the destruction of collagen and elastin. MMPs are a type of proteolytic enzyme whose activity is thought to be augmented in AAA, as evidenced by analysis of human aneurysmal tissue (14, 15). The increase in MMPs and a decrease in tissue inhibitors of MMPs lead to the break down of collagen and elastin in the ECM as well as VSMC depletion, resulting in degradation of the ECM. This leads to thinning and destruction of the normal aortic wall architecture, predisposing the individual to dilational changes and aneurysm development (8, 16–18). Apoptosis of VSMC characterised by inflammatory cell infiltrate, reactive oxygen species (ROS), and endoplasmic reticulum stress with the degeneration of aortic media, are the hallmark of AAA pathology (19).

Data related to the mechanism of TAA represent it as more than just a degenerative process, but rather a multifaceted culmination of both intracellular and extracellular alterations. It is characterised by abnormalities in ECM that compromise the structural integrity of the aorta (19).

## Diabetes Mellitus Impacting Aortic Aneurysms

### The Epidemiological Picture

DM, a chronic metabolic disorder characterised by hyperglycemia, insulin resistance and/or deficiency (relative or absolute), is associated with various microvascular and macrovascular complications contributing to major morbidity and mortality (20). DM is a significant risk factor for cardiovascular disease and reports have shown an inverse relationship between the disease and both AA prevalence and incidence (21, 22).

An ultrasound screening done in a regional veterans health care system as part of the Aneurysm Detection and Management study showed a lower prevalence of AA in the diabetic group. A significant number of other epidemiological studies have also confirmed the negative association of DM and AA, suggesting it to be protective in the formation and expansion of AA (23). A paradoxical inverse relationship was observed between the severity of DM and AA and also a reduced prevalence was seen in an Asian population study (24). Experimental studies in animal models also show attenuation of AA development in the presence of hyperglycemia.

The Viborg Vascular randomised screening trials of Central Denmark showed an inverse relationship between AA growth rate and HbA1c concentration (25). Certain retrospective studies have also observed a realistic association between DM and AA indicating a diabetic subset less likely to have an aneurysmal rupture and death (26). Another epidemiological study investigated the comparison of age at rupture and showed diabetics under the age of 65 had no aneurysmal rupture. Conversely, 15% non-diabetics developed rupture under the age of 65 years (26, 27). Other studies showed the growth rate of smaller AA and associated expansion much slower than in non-diabetics, again suggesting a negative association (28).

Furthermore, the effect DM has on the outcome of AA repair revealed no significant difference in the morbidity and mortality (29, 30), whereas a few other studies showed lower mortality rates in the diabetic population (31). Complications associated with open AAA repair can include myocardial infarction, pancreatitis and infections such as pneumonia; higher rates of these complications were observed in the diabetic population post-operatively (30).

### Impact on TAA

TAA in the vast majority is asymptomatic and diagnosed *via* echocardiography or CT performed for other indications, until a catastrophic event like AD-related complications occur, such as cardiac tamponade, acute aortic regurgitation or stroke (32). A negative correlation between DM and TAA formation and growth rate was also found. Additionally, a case study analysis showed the prevalence of DM in TAA was significantly lower (8). Prakash et al. (33) found an inverse association between DM and hospitalisations due to TAA. Also, those with DM complications had the lowest rate of TAA. Hyperglycemia, which is associated with reduced adventitial neovascularisation and decreased inflammatory cell infiltration in the medial layer of the aorta, probably inhibits progression of TAA by reducing VSMC death and ECM degradation (33). A Spanish study involving Spanish national data revealed a higher indicidence of TAA in the non-diabetic population compared to the diabetic population. Additionally, the mortality associated was significantly lower in the diabetic than the non-diabetic population (34).

### Impact on AAA

AAA have increased in the past two decades adding substantial burden to the healthcare of developed countries. They are often asymptomatic, being detected incidentally *via* routine imaging or as a medical emergency in case of rupture (3, 4). Multiple studies were done to determine the impact of DM on the incidence, growth rate, prevalence, morbidity and mortality of AAA.

De Rango et al. (35) and other prospective studies showed significant decrease in the incidence of new AAA in a diabetic population. Takagi et al. (36) assessed the growth rate in both diabetic and non-diabetic groups and established that DM is associated with a reduced rate of AAA growth. Reduced aneurysmal growth rate and expansion was also confirmed by Vega et al. (27). The life threatening complication of aneurysmal rupture and the associated mortality was also found to be lower in diabetics than non-diabetics (26, 28). The association was also more significant in diabetic males than females (36, 37).

Golledge et al. demonstrated the role of DM and glycation on AAA expansion: DM was associated with slower progression of AAA. The glycated ECM was found to markedly inhibit monocyte MMP production thus explaining the protective role of DM on AAA. There were lower levels of IL-6, MMP-2 and MMP-9 being secreted in the glycated ECM (7). On the contrary, a higher myocardial infarction and wound infection rate in the first 30 days was observed in diabetics with AAA by Treiman et al. Also the long term survival was found to be lower in diabetic patients adding to the increased cardio-vascular burden in a series of studies (35, 38, 39).

A study in the Asian population revealed significant reduction in association of advanced and severe Type 2 DM (T2DM) with AAA without rupture and also a decreased prevalence of AAA in the diabetic population (24). Dua et al. demonstrated an increase in the PAI-1 level with decreased plasmin and thus a low MMP-2 and MMP-9 levels in the DM-AAA induced mice population. This reinforces the protective role of hyperglycemia might have on aneurysm formation and aortic diameter (40). A study by Miyama et al. (41) investigated the role hyperglycemia has on aneurysm progression and found reduced levels of macrophage infiltration, neovessel density and MMP-9 levels, thus conferring protection against aneurysm formation.

Data surveillance from CAESAR, a multicentre randomised trial that compares the efficacy of surveillance vs. endovascular aortic repair in small AAA, by De Rango et al. compared diabetics and non-diabetics. Though the aneurysmal growth rate in the first year was similar in both the groups, later increase was higher in the non-diabetic group. Patients with DM in the surveillance group required lower rates of endovascular aortic repair after 30 months but no significant difference in adverse events between diabetics and non-diabetics was observed (35).

### Impact on AD

Acute Aortic dissection is relatively uncommon and first described over 200 years ago (42). AD involves a tear of the aortic intima, resulting in the formation of a false lumen, with inflow of blood in to the medial layer. The consequential false lumen may propagate distally or even retrogradely making it an aortic emergency (43). The pathology involves acute separation of layers within the aortic wall following an initial intimal tear. Luminal blood enters the intima-media layer creating a life threatening condition requiring immediate assessment and management with 20% patients dying before they reach the hospital and 30% during the hospital admission. Patients with enlarged AA, carry with them a risk of 10% per-year of sudden death as a result of the occurrence of AD. Hypertension, substance abuse, connective tissue disorders, family history of thoracic aortic diseases, vascular inflammation, congenital disorders are all risk factors for the occurrence of AD (43).

Data from inpatient sampling to determine the association between AD and DM established that DM was significantly and negatively associated with TAA and AD; this was found to be independent of multiple clinical characterisitcs and variables, including: type of hospital, the region, age of the patient and income. Hospitalisation due to TAA and AD occurred at a lower rate in diabetics (33). The achieved results in the literature that illustrated this inverse association was seen in both men and women, appearing to be strongest amongst those with diabetic complications. Therefore, the aforementioned suggests that the inverse relation of DM with TAA and AD may further be influenced by the severity and duration of hyperglycemia, as well as the level of susceptibilty a patient has to vascular injury. A nationwide observational study comparing T2DM patient group with a control group, reported a lower short term mortality post-hospitalisation, reaching upto greater results at 2-years with better survival rates for the diabetes group (33). Alteration of aortic tissue through systemic cross linkages contribute to the protective effect of diabetes toward stabilisation of the aorta, preventing dilatation, growth and rupture (44).

A non-western population case control study performed to assess the correlation between AD risk and DM also concluded that DM was significantly associated with a decreased risk of AD. However, it is important to note that the authors reported no significant difference in in-hospital mortality when comparing the DM and non-diabetic patients (45).

## Overcoming a Challenge: the Use of Animal Models

It is challenging to define AA mechanisms in humans, which commonly results in the use of animal models to gain a deeper understanding of the pathophysiology. Although it is difficult to obtain the exact pathophysiology using animals, several probable mechanisms have been studied addressing the deficiencies in animal models (46). Dissecting and non-dissecting AAA models are used, with the non-dissecting AAA created through calcium chloride or porcine pancreatic elastase (PPE) perfusion.

There is evidence of reduced expression of Cell Division AutoAntigen (CDA-1) in the aortic biopsies of human AAA. The CDA-1 which enhances transforming growth factor (TGF)-β signalling is found to be upregulated in diabetes and is also found to play a key role in the protective effect conferred by DM on AA (47). In a study by Jiaze Li et al., DM was induced in male wild-type CDA-1 knockout (KO), Apolipoprotein E (ApoE) KO and CDA-1/ApoE double KO mice. This model was characterised by Angiotensin II (Ag-II) induced aneurysms, and the results demonstrated that CDA-1 lacking mice with diabetes developed aneurysms. On the other hand, with CDA-1 present, the severity of these aneurysms was reduced in diabetic mice and was characterised by reduced fatal aortic rupture and attenuated supra-aortic expansion (47).

The study by Miyama et al. involved hyperglycemic and euglycemic murine (mice) models. Through intra-aortic PPE infusion or by Ag-II subcutaneous infusion, the formation of AA was induced in the mice. To assess the effect of decreasing serum glucose levels, insulin therapy was also instituted in a separate cohort. The study showed hyperglycemia was linked with reduced mural neovascularisation, macrophage infiltration, and medial elastolysis. Also insulin mediated glucose reductions did partially negate the protection rendered by hyperglycemia on AA (41).

Calcium phosphate can contribute to aneurysm formation by causing apoptosis of VSMCs with subsequent macrophage infiltration. The study done by Tanaka et al. was on Type 1 and Type 2 Diabetic mice (murine models) and in them calcium phosphate induced aneurysm formation was found to be significantly suppressed in the presence of hyperglycemia. The mechanism was found to be through suppression of macrophage activation and aneurysmal degeneration through the activation of Nr1h2 (liver X receptor α and β) (48).

## Diabetes, a Protective Factor? Potential Mechanisms Leading the Way

### Wall Stress

In comparison to other arteries, the aortic wall suffers from much greater stress. A typical ageing process is aortic dilation, which occurs in about a quarter of otherwise fit and healthy patients. Although the aortic wall is thicker alongside increasing age, wall stress autoregulation seems to be faulty in males, implying that the aorta is an artery susceptible to damage. Compared to non-diabetics, the AAA expansion rate is at 30%, which is not a substantial difference. An altered remodelling response observed in diabetic patients with AA compared to healthy subjects leads the protective response of AA in DM (49–51).

One of the critical factors in causing and enlarging AAA is aortic wall stress. However, diabetics are known to have a larger matrix volume leading to thicker aortic walls, which reduce aortic wall stress (7, 49, 52). Recent studies show the hyperglycemia associated with DM plays a significant role in stabilising the collagen network through crosslinking of collagen in the aortic wall media (7, 49).

### Matrix Metalloproteinases

MMPs are secreted *via* endothelial cells and macrophages, which exhibit increased activity, through proteolytic action in human aneurysmal tissue and are activated by wall stress. Specifically, through the enzymatic process of disintegrating various proteins such as elastin and collagen within the vessel wall, both MMP-2 (released *via* smooth muscle) and MMP-9 (released *via* macrophages) are involved in destructing the matrix. It has been observed that both MMP-2 and MMP-9 are increased in patients with AAA, whereas mice lacking these enzymes do not develop any dilatation of the aorta (53–57). This may be the potential mechanism through which diabetic patients have a preserved aortic matrix- due to decreased MMP activity. A lower concentration of MMP-1, MMP-2, and MMP-9 has been observed by a study that investigated the differences in MMP activity and presence in both diabetics and non-diabetics (58). Hence, it can be concluded that the potential mechanism for reduced aneurysmal events in diabetics may be through reduced MMP activity primarily, through the effects of high glucose levels (21, 49, 50).

### ECM Remodelling, Glycation, and Advanced Glycation End Products

The vascular ECM contributes to the cellular structure and tissue organisation, which comprises a vast system of components that include: elastin, basement membrane, collagen and proteoglycans (6). The interaction of the ECM and the various cells of the arterial wall play an important role when it comes to vessel remodelling. An example of this cellular remodelling can be seen in diabetic patients, with the advanced glycation of ECM proteins such as collagen, as a result of hyperglycemia. A crucial step involved in the formation and progression of AA is the secretion of MMPs, which result in proteolysis. The crosslinking of collagen and elastin resultant of the advanced glycation of the ECM within the aortic wall of the abdomen, results in the inhibition of MMP secretion and subsequently the aforementioned mechanism (6, 7).

Polysacchiride glycosaminoglycans are a component of the ECM by covalent bonding with the existing proteins forming proteoglycans. There is an abundance of biglycans in the normal aorta, however, it is decreased in the setting of AAA. Biglycans can regulate TGF-beta signalling pathway and *vice-versa*. This implies the presence of regulation *via* a mutual positive feedback mechanism, allowing the ECM's preservation, thereby providing protection against the progression of AA. The direct impact of DM on biglycans is uncertain; there may be the augmented pathway of the TGF-β signalling caused by upregulation of CDA-1 (59, 60). Furthermore, the physiological process of other glycosaminoglycans in the aorta, involving deposition, production and degradation, can be affected by the presence of DM (61).

DM and chronic hyperglycemia lead to advanced glycation end products (AGE), which influence the activation of monocyte-macrophages. This is achieved *via* unique receptors for AGE (RAGE), the engagement of which results in AGEs bringing about non-enzymatic crosslinking between ECM basement membrane components (6, 7). These mechanistic processes add to the pathophysiology of atherosclerosis, and result in numeorus signalling pathways being stimulated. It thus contributes to arterial stiffness, protecting against mechanical structural loss and resists proteolysis. The distinct properties of ECM in diabetic patients confer a protective effect on AAA (6, 7, 14, 62, 63).

### The Role of Inflammation

Inflammatory processes have a vital role in AAA and have a significant influence on many of the factors of remodelling of the wall of the aorta (16). The role inflammation plays in the negative association of DM and AAA is complex (16). The exact mechanism through which DM influences inflammation in AAA might include the stimulation of T-cell insulin receptors, the monocyte-macrophage system or through C-peptide production (41). Patients with T2DM often have increased C-peptide levels.

A study by Cifarelli et al. demonstrated that physiological levels of C-peptide can decrease hyperglycemia induced VSMC proliferation (41, 64). Haidet et al. investigated the effect of C-peptide presence on monocyte cell lines surrounded by a glucose solution. The study demonstrated a decreased expression of multiple pro-inflammatory cytokines through the NF-KB mediated pathway in the presence of C-peptide (65, 66). Studies in mice have shown low levels of IL-6 restricts both TAA and AAA progression. Glycation additionally has the potential to alter monocyte-macrophage function toward an anti-inflammatory phenotype which decreases IL-6 production (7). Additionally, Mendelian randomisation approaches support the involvement of IL-6 receptor pathway in human AAA. Glycation of IL-6 could be another factor contributing to the protective effect of DM on AA (67).

### Aortic Mural Neoangiogenesis

An important element in pathophysiological processes for aneurysms, and potential rupture, is the role played by aortic mural neoangiogenesis. In the event of AAA, there is often the linked formation of a mural thrombus; the thrombus undergoes continuous remodelling as a result of the maintained blood flow throught the abdominal aorta. The thrombus can significantly lower the wall stress, there is the impeding influence of the greater wall thickness. This structural change can subsequently result in the inner aspect of the media to be exposed to hypoxic conditions locally, which consequently leads to inflammatory response and augmented medial neovascularisation (68).

In hyperglycaemic mice, it has been found that the reduced AA diameter is concomittant with lower levels of medial elastolysis, macrophage infiltration, in addition to lower level of this neoangiogenesis. In murine models, hyperglycemia inhibiting neovessel formation by down regulation of activation of vascular endothelial growth factor expression and angiogenic response was observed. Beyond neovascularisation and macrophage infiltration, additional mechanisms include hyperglycaemic influence on the fibrinolytic system, RAGE and progenitor cell function (41).

### Intra-luminal Thrombus

Intra luminal thrombus (ILT) implicated in the pathogenesis of AA, has high concentration of MMP-9 within the thrombus and signs of collagenolytic activity. During the process of thrombus renewal, MMPs that can be found inside the thrombus are released through various processes including fibrinolysis (69–71). Dunn et al. (72) showed the clots in diabetes are denser and lower level of susceptibility to fibrinolysis.

T2DM is often associated with hyperinsulinemia which could also have a protective influence by preventing ILT renewal. Hyperinsulinemia increases PAI-1 levels and inhibits plasmin being converted from plasminogen, which decreases fibrinolysis. This in turn decreases expression of MMP, as plasmin is needed to convert proMMP to its active form (73–75). These fibrinolytic changes and renewal potential reduce clot degradation of ILT in AAA. Stability of the aortic wall can be improved through this along with reducing the rate at which the aneurysm expands (71, 72).

### Vascular Smooth Muscle Cell Homeostasis

Another vital component of the pathophysiological processes involved in the development of an aneurysm are the VSMC. Most VSMC are of contractile phenotype contributing to the vascular tone. These cells play a key role in vascular remodelling due to their ability to differentiate their phenotype, to a synthetic form. Synthetic phenotype switching triggered by oxidative stress, inflammation and injury is characterised by decreased contractile protein expression and increased MMPs. The synthetic phenotype also leads to calcification, in turn leading to risk of aneurysmal progression and rupture. AA is characterised by disrupted vessel wall structure with characteristic histology of VSMC apoptosis. TAA and AAA share a common feature of VSMC depletion and ECM proteolysis (76).

**Figure 1:**
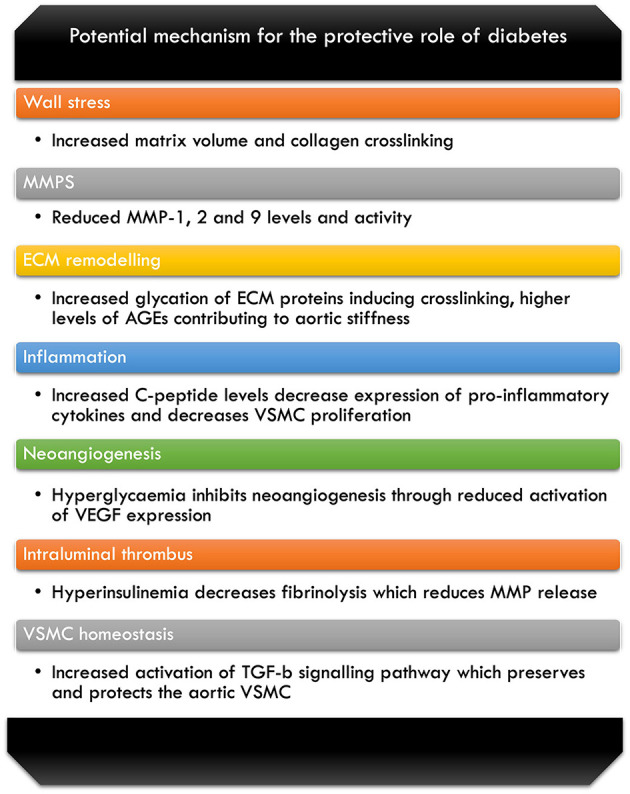
Breakdown of some of the potential mechanism for the protective role of diabetes against AA disease (6, 7, 14, 41, 49, 52, 58, 63–66, 69–72).

TGF-β is required for inducing and maintaining the homeostatic and differentiating processes of the VSMC, for the mechanisms discussed. The environment created as a result of DM causes greater stimulation of the TGF-β signalling pathways, which has local and systemic effect on the VSMC of the aorta, hence plays an important protective role (6). Furthermore, as the hyperglycaemic state in the DM patients influences the VSMC, it contributes to the protection against development of aneurysm and AD through its homeostatic actions in the wall of the aorta (6) (Figure 1).

## Hypoglycaemic Agents: the True Heroes?

A number of factors, in DM patients, must be controlled to maintain a tight glycaemic control that is dependent on various medical regimes—these aim to reduce the severe risks associated with DM. Apart from a patient centric approach with life style modification, involving dietary changes and regular exercise regimens, the effective management of diabetes also involves choosing appropriate pharmacological treatment for the blood glucose control. The anti-diabetic medications used include Biguanides, SGLT2-inhibitors, dipeptidyl peptidase (DPP4) inhibitors, thiazolidinediones, glucagon-like-peptide (GLP)-1 receptor agonists, sulfonylureas and insulin therapy (77).

Various studies of different experimental designs have concluded that drugs used to manage DM have a protective role against AA.

### Biguanides—Metformin

Metformin is one of the oldest first line oral hypoglycaemic agent used in DM management. The mechanism of action of Metformin involves a reduction in the hepatic glucose production and also improvement in the tissue sensitivity to insulin. It has pleiotropic anti-inflammatory and vasculoprotective effects through various mechanisms including limiting aortic inflammation, reduction in the ECM remodelling and decreasing the oxidative stress (57, 78).

Studies by Golledge et al. and a nationwide analysis of of Veterans Affairs with DM also showed that Metformin did limit the growth of AAA (79, 80). Mice experiments by Fujimura et al. showed that the administration of Metformin during both aneurysm induction and progression periods reduces the initiation and progression of AAA in non-diabetic mice. The Metformin mediated resistance to AA was associated with reduced inflammation, elastin degradation and VSMC depletion in the histopathologic examination (81).

An improvement in the aortic elasticity was observed in a study by the addition of Metformin to oral contraceptive pills (82). Vasamsetti et al. (83) demonstrated in an Ag-II treated mice that the presence of Metformin reduced the proinflammatory cytokine levels indicating the protective role it has against aneurysm formation.

*In-vitro* studies have shown Metformin additionally reduces MMP-2 and VSMC proliferation in human aortic cells. Additionally, lower incidence of AAA associated events were found in diabetics on Metformin compared to those not on Metformin (14).

### PPAR Gamma Agonists—Thiazolidinediones

Thiazolidenediones include Pioglitazone and Rosiglitazone and these drugs act as PPAR-γ agonists and exhibit their anti-inflammatory effects by lowering tumour necrosis factor (TNF)-α levels.

Motoki et al. studied the effect of PPAR-γ agonists on the aortic wall in patients with AA and found that it may prevent or delay the progression of the aneurysm in patients *via* decreasing the expression of MMP-9 and TNF-α in the wall of the AA (84).

Piranov et al. investigated in ApoE deficient mice, the mechanism of Rosiglitazone in the earlier phases of AAs induced by Ag-II, looking at potential sites at which the drug would act. Rosiglitazone worked by inhibiting c-Jun N-terminal kinase phosphorylation and reduces the regulation of toll-like receptor 4 (TLR4) being expressed at the location at which the lesion is being formed during the initiation stage of experimental AA development. The authors further discussed an additional mechanism *via* which the initiation stages of aneurysm formation is blocked. They explain the relation of the drug with lowered levels of MIP-1α and MCP-1 chemokines, which are pro-inflammatory, as well as declining the level of CD4 antigen (85).

The action of PPAR ligands were studied by Golledje et al. investigating their impact on AA in mouse models. This study reported a significant association of Pioglitazone with lower expansion of the suprarenal aorta. This linkage may potentially be due to down regulation of osteopontin, a chemotactic factor which has an association with AAA in humans (86).

Rosiglitazone could also exert its protective effect on AAA development by reducing serum levels of MMP-9 in individuals with T2DM and thus playing a role in ECM preservation (87). Jones et al. showed that Rosiglitazone led to less inflammation due to the lower amounts of mediators IL-6 and TNF-α. Furthermore, the use of the drug makes the ECM thicker, as a result of its associated amplified production of collagen, thus reducing chances of rupture and death (88).

### Dipeptidyl Peptidase Inhibitors

DPP4 inhibitors like Sitagliptin, Vildagliptin, Teneligliptin and Alogliptin act by inhibiting the proteolytic enzyme DPP4 thus prolonging the action of GLP and delaying gastric emptying, improving glycemic control by increasing meal stimulated insulin secretion and inhibiting glucagon release.

Boa et al. studied the protective effect of DPP-4 inhibitor, Alogliptin. The study investigated on rat AAA models and found that the drug attenuated the formation and expansion of aneurysms in a dose-dependent manner *via* an antioxidative action. Rats on alogliptin showed lower rates of reactive oxygen species (ROS) activity, thus inhibiting aortic wall destruction. A reduction in the MMP-2 and MMP-9 levels were also observed. A significant reduction in aortic dilatation in the DPP-4 inhibitor-treated rats compared to the control group was observed in macroscopic findings (89).

Lu et al. studied the protective effect of Sitagliptin in induced AAA mice models. The incidence of AAA in Sitagliptin treated mice was much lower, around 4–8%. Sitagliptin-treated models showed attenuation of elastin and collagen disruptions with decreased MMP-9 and MMP-2 activity, substantial reduction in macrophage infiltration and reduced apoptosis in the wall of the aorta, thus indicative of a potential protective role of sitagliptin in AA formation (90).

### Sulfonylureas

Sulfonylureas are one of the commonly used potent oral hypoglycaemic agents. A case control analysis done by Hsu et al. with the use of Taiwan's national health insurance research database concluded that diabetic patients on oral hypoglycaemic agents, including sulfonylurea, biguanides and thiazolidinediones were associated with decreased risk of AA development. The exact mechanism by which sulfonylureas exert their protective effect is not known, as no direct clinical or experimental studies are available in this specific area.

The probable mechanism may be through the SUR-2 receptor mainly expressed on the VSMC wall (91). Indirect evidence for the aforementioned was suggested in a study by Hiraki et al. (92) reporting on a patient with AA from a family with Cantu Syndrome, a genetic disorder characterised by ABCC9 mutation affecting both the SUR-2A and SUR-2B.

### GLP-1 Receptor Agonists—Lixisenatide

Yu et al. demonstrated the protective effect of subcutaneous Lixisenatide on development of an aneurysm. The use of Lixisenatide resulted in reduction in ROS and macrophage infiltration; additionally, there is inhibition of the expression of the genes for MMP9, TNF and MMP2 in the walls of the aorta of the experimental rats. These processes instigates the protective action that Lixisenatide has *via* anti-inflammatory and anti-oxidant actions, as well as preserving ECM (93).

In Ag-II infused ApoE KO mice, Liraglutide administration was found to reduce the formation of AAA, as discussed by Lu et al. (90). This was achieved by increasing the circulating active form of GLP-1, reducing the infiltration of macrophages, and decreased expression of MMP-2 and MMP-9, thereby preserving the elastin content (90).

### SGLT2 Inhibitors—Showing Promise?

SGLT2 inhibitors are a more recent hypoglycaemic agent, which inhibit the action of the SGLT2 proteins responsible for the reabsorption of the majority of the glucose that has been filtered. These proteins are situated at the proximal convoluted tubule. The action of these drugs is *via* the blockade of the respective low affinity and high capacity proteins. As a result of this mechanism of action these drugs cause glycosuria, consequently lowering the patients plasma glucose concentration in an insulin and incretin pathway independent manner (94–96). The commonly used drugs of this class are Canagliflozin, Dapagliflozin and Empaglifozin. Apart from the kidneys, the SGLT2 protein is also found to be expressed in other tissues including: adipose tissue, vascular tissue, such as endothelial cells, and the aortic wall (97–99). Therefore, SGLT2 inhibitors have been found to have pleotropic effects, most importantly cardio-protective effect along with their glucose lowering action.

Ortega et al. demonstrated that chronic oral Empagliflozin use reduced the Ag-II induced supra-renal AAA development in ApoE KO mice. The attenuation was a result of reduction in macrophage infiltration within the lesion and down-regulation of pro-inflammatory cytokines that was observed with Empagliflozin administration (100). SGLT2 inhibitors were additonaly seen to have an impact on lowering the levels of MMP-9 and MMP-2, along with reduced atherosclerosis, in ApoE KO mice. The authors also observed a marked reduction in vascular endothelial growth factor levels and neovascularisation in these Empagliflozin co-treated mice. This cotreatment was found to diminish Ag-II induced elastin degradation in immunohistochemistry analysis (100).

*In-vitro* studies in human aortic endothelial cells revealed that Empagliflozin decreased mononuclear-leucocyte endothelial cell interactions and endothelial production of chemokines induced by Ag-II (100). Kaji et al. (101) have also demonstrated *in-vitro* suppression of human endothelial cell proliferation and tubular formation by Canagliflozin. Thus, chronic oral administration of SGLT2 inhibitors show promise as novel glucose lowering agents, displaying pleotrophic effects on AAA. Further research in this field will add greater value to its therapeutic use in DM and beyond.

### What About Insulin Therapy?

The oral hypoglycaemic agents have shown to confer a protective effect on the incidence and progression of AA. Improvement of hyperglycemia by insulin therapy however, has been found to negate the protective effects (41).

## Future Perspectives

DM is an established cardiovascular risk factor and accelerates atherosclerosis. Experimental studies and data from epidemiological studies have shown DM to have a protective effect against the development, growth, expansion and rupture of AA. Human and animal studies have shown a spectrum of mechanisms by which DM could exert a protective effect on AA and dissection. Additionally, medications used in the management of hyperglycemia have shown to independently confer a protective effect on the formation and progression of AA.

These observations could help identify high risk groups to help in the primary prevention of AA. Understanding the pathogenesis involved helps in developing future prevention and management strategies both medically and surgically. The evidence of reduced expression of CDA-1 in the human AA walls and the protective effect of CDA-1 seen in experimental mice enables us to plan further involving CDA-1 as a potential therapeutic target in the prevention and management of AA. The downregulation of MMPs observed in animal models, as a potential mechanism involved in the protection observed in diabetes and with oral hypoglycaemic agents, could also act as a potential therapeutic target in future studies.

Further investigations are still needed to establish the mechanism implicated in the beneficial effect of diabetes on AD. Detailed studies into the cellular and molecular mechanisms and other genetic factors contributing to the aneurysm disease process would be required to individualise the treatment strategies in future.

## Conclusion

DM has a significant impact on the healthcare of our society and has clearly been shown to be a risk factor for cardiovascular disease. However, our review of the literature has demonstrated the reported protective effects that DM has in the development and progression of AA, as well as with AD.

The literature has reported various mechanisms by which hyperglycemia and presence of DM exerts its protective effects; however, it is important to note that there is yet to be a definitive consensus reached for all the proposed mechanisms. Figure 1 illustrates these potential mechanisms against AA disease that we have covered in our review of the literature.

Building on the aforementioned, our review has highlighted the impact of oral hypoglycaemic agents themselves playing a role in the protection against AA disease. Table 1 provides a detailed overview of the classes of drugs that we have discussed, as well as covering the negating action of insulin therapy on the protective effort by the discussed oral hypoglycaemic agents.

**Table 1:** A brief outline of the mechanism by which various hypoglycaemic agents have a protective impact against AAs.

**Hypoglycaemic agent**	**Mechanism of aortic aneurysm prevention**
Biguanides–Metformin (14, 80–82)	• Resistance to AA through: reduced inflammation, elastin degradation and smooth muscle cell depletion
	• Combined with oral contraceptive pills, increased aortic elasticity is observed
PPAR gamma agonist–Thiazolidinediones (84–86, 88)	• Decreased expression of TNF-a, MMP-9, and IL-6 in AA wall but increased collagen production
	• Inhibition of JNK phosphorylation and down-regulation of TLR-4 expression associated with reduced proinflammatory chemokines
	• Reduced aortic expansion due to downregulation of osteopontin
Dipeptidyl peptidase inhibitors (89, 90)	• Dose dependent suppression of ROS
	• Reduced levels of MMP-9 and MMP-2
	• Reduced apoptosis in wall of the aorta and reduced macrophage infiltration
Sulfonylureas (91, 92)	• Associated with lower risk of AA development
	• Exact protective mechanism is not known
	• Proposed mechanism involves action of SUR-2 receptor on VSMC (9) wall.
GLP-1 receptor agonists-Lixisenatide (93)	• Reduction in ROS and macrophage infiltration
	• Inhibition of TNF-a, MMP-2 and MMP-9 gene expression in the aortic wall
SGLT2 inhibitors (94–100)	• Chronic use demonstrated reduced AAA development
	• SGLT2 Inhibitors have a pleotropic effect, with cardio-protective effects seen as well as just for DM treatment
Insulin therapy (41)	• Insulin therapy negates protective effects of oral hypoglycaemic agents

*AA, aortic aneurysm; TNF-a, tumour necrosis factor-alpha; MMP, matrix metalloproteinase; IL, interleukin; JNK, C-Jun N-terminal kinase; TLR, toll-like receptor; ROS, reactive oxygen species; VSMC, vascular smooth muscle cell*.

There is the need for further research into the association of DM and AA, investigating the molecular and genetic aspects with understanding of the natural history of these diseases. This will allow greater development of decision-making framework for management as we aim toward precision medicine for our patients (Table 2).

**Table 2:** A summary of the key points from the review of the literature discussing the impact of DM on AA disease, and its role as a protective factor.

**Summary table**
AA and DM have a well-known significant disease burden
DM is a recognised risk factor for cardiovascular disease; however, literature suggests a protective role of DM in AA, thoracic and abdominal, with this association being observed with aortic dissection as well
There have been numerous mechanisms proposed for the protective role of AA in DM, these are related to: reduced wall stress, inflammation, neoangiogenesis, lower matrix metalloproteinases, and increased stimulation of transforming growth factor-beta providing protection for the vascular smooth muscle cell
Clinical and experimental studies have reported that the drugs used in the management of DM have a protective role against AA
Further research, including molecular and genetic studies, into the link between DM and AAs could help with individualising treatment strategies in the future

*AA, aortic aneurysm; DM, diabetes mellitus*.

## Author Contributions

DA, WM, LS, RV, JR, MB, IW, BV, and MI have all collectively contributed to the intellectual property of this article, and have been involved in design, structure, data collection and revisions of this article. All authors contributed to the article and approved the submitted version.

## Conflict of Interest

The authors declare that the research was conducted in the absence of any commercial or financial relationships that could be construed as a potential conflict of interest.

## Publisher's Note

All claims expressed in this article are solely those of the authors and do not necessarily represent those of their affiliated organizations, or those of the publisher, the editors and the reviewers. Any product that may be evaluated in this article, or claim that may be made by its manufacturer, is not guaranteed or endorsed by the publisher.
